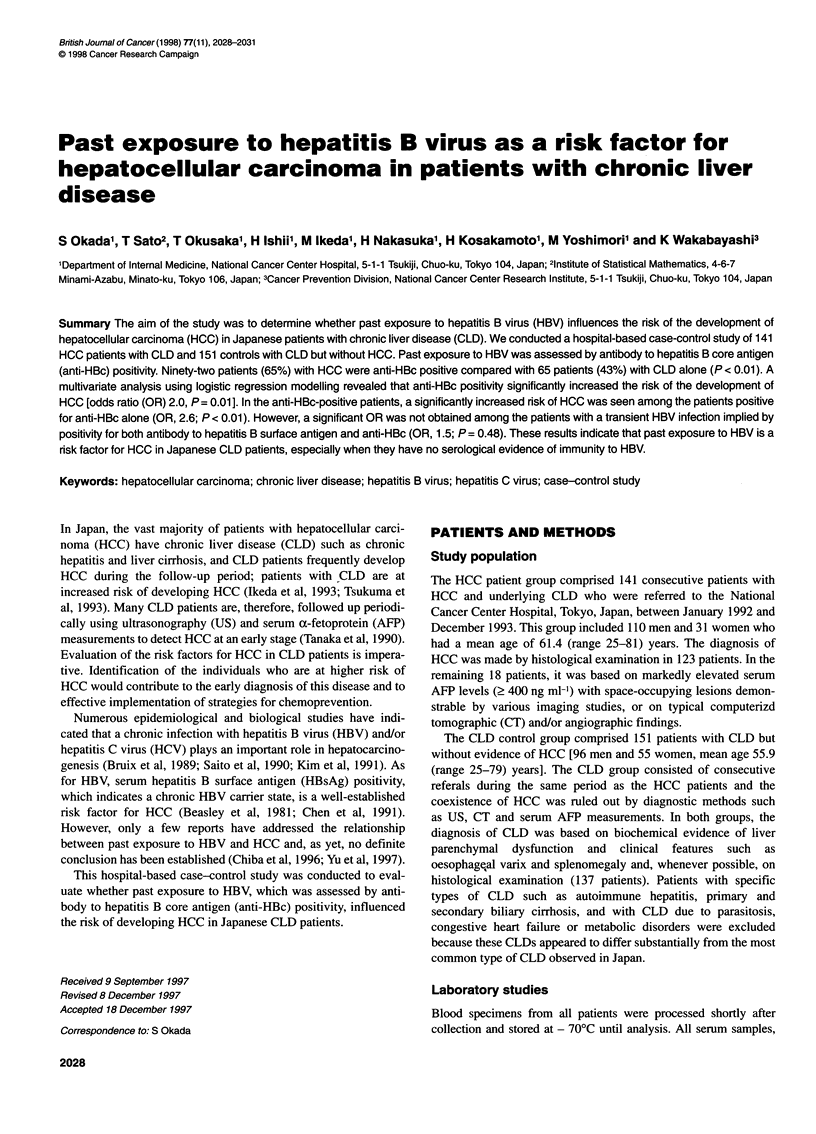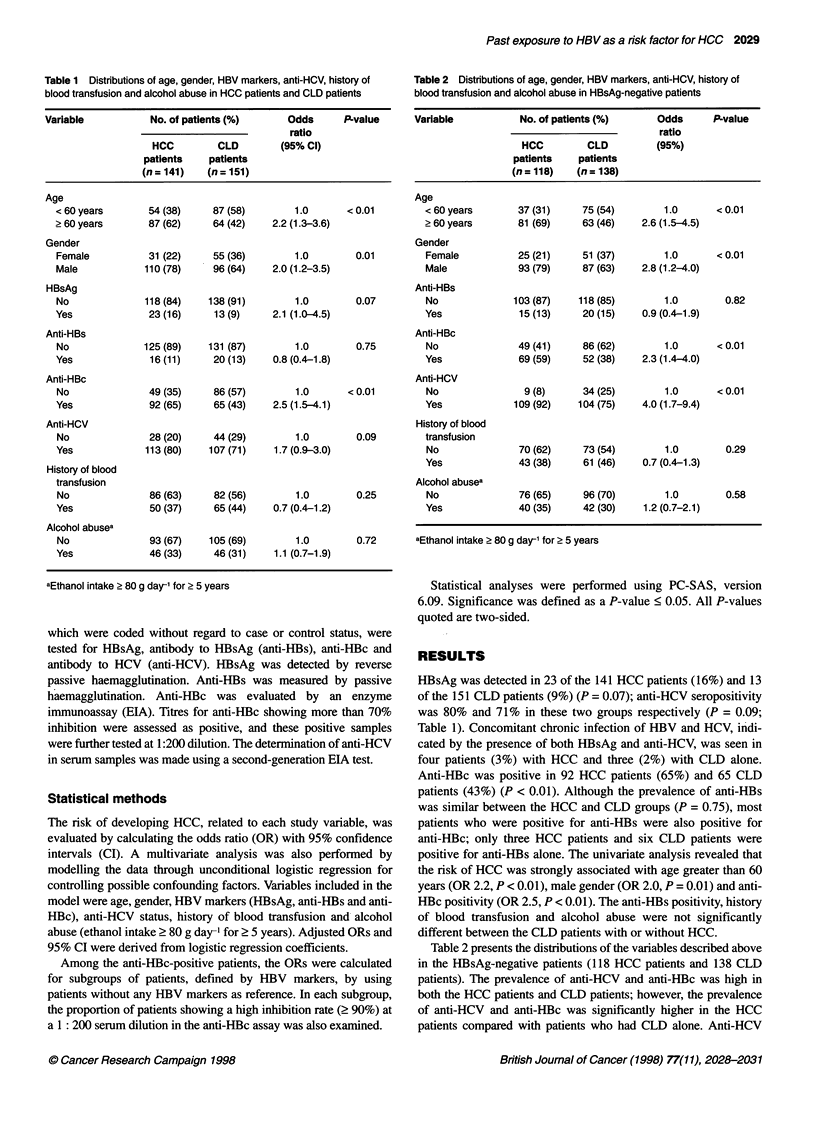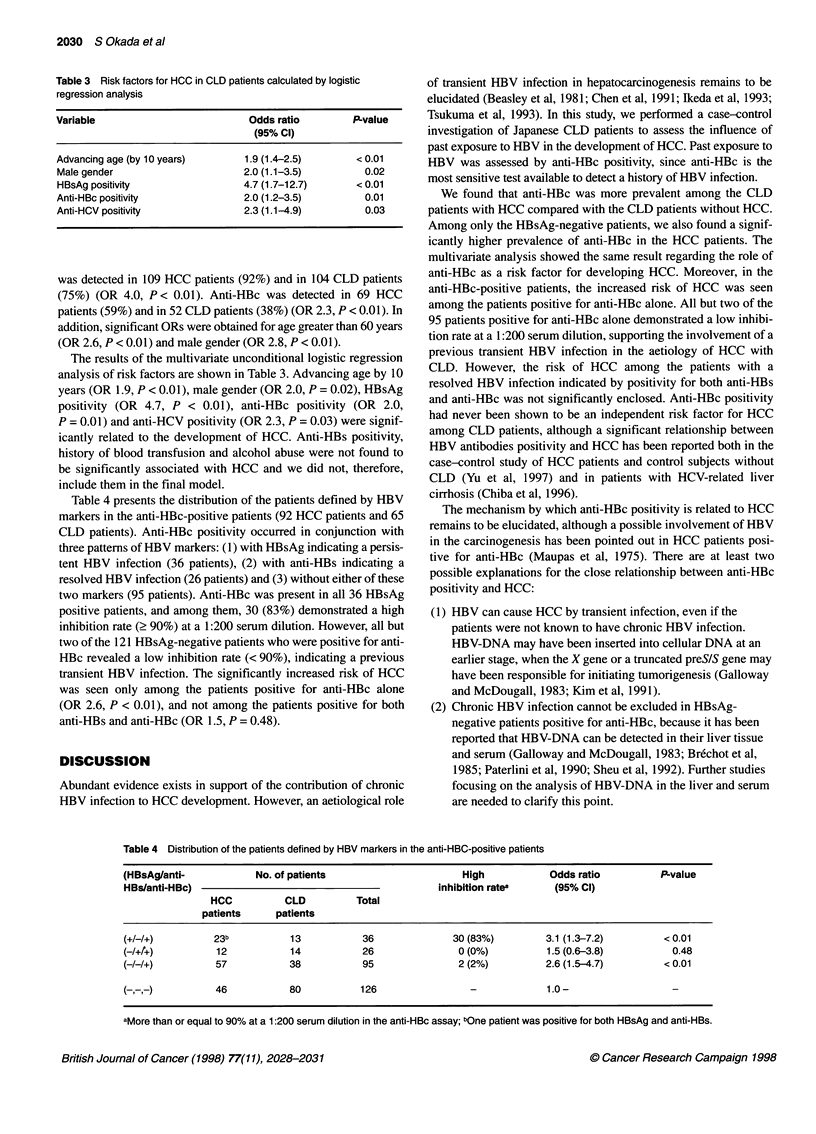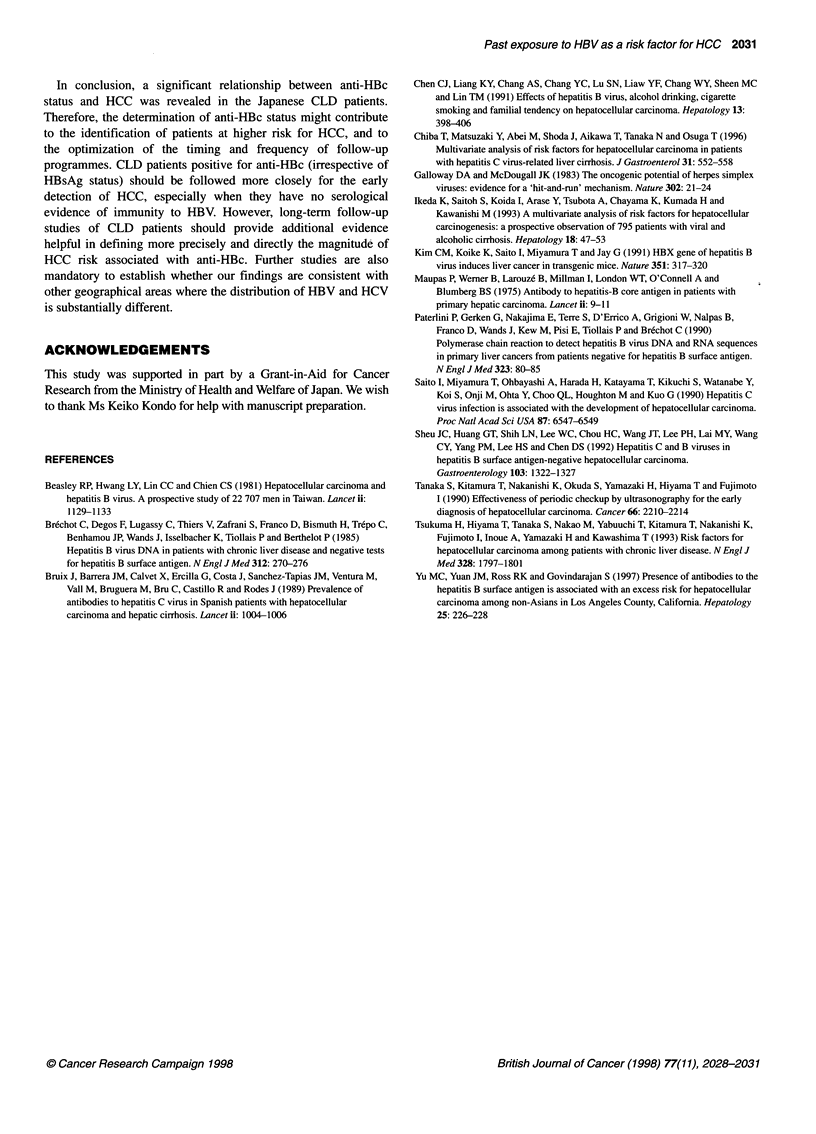# Past exposure to hepatitis B virus as a risk factor for hepatocellular carcinoma in patients with chronic liver disease.

**DOI:** 10.1038/bjc.1998.337

**Published:** 1998-06

**Authors:** S. Okada, T. Sato, T. Okusaka, H. Ishii, M. Ikeda, H. Nakasuka, H. Kosakamoto, M. Yoshimori, K. Wakabayashi

**Affiliations:** Department of Internal Medicine, National Cancer Center Hospital, Tokyo, Japan.

## Abstract

The aim of the study was to determine whether past exposure to hepatitis B virus (HBV) influences the risk of the development of hepatocellular carcinoma (HCC) in Japanese patients with chronic liver disease (CLD). We conducted a hospital-based case-control study of 141 HCC patients with CLD and 151 controls with CLD but without HCC. Past exposure to HBV was assessed by antibody to hepatitis B core antigen (anti-HBc) positivity. Ninety-two patients (65%) with HCC were anti-HBc positive compared with 65 patients (43%) with CLD alone (P < 0.01). A multivariate analysis using logistic regression modelling revealed that anti-HBc positivity significantly increased the risk of the development of HCC [odds ratio (OR) 2.0, P = 0.01]. In the anti-HBc-positive patients, a significantly increased risk of HCC was seen among the patients positive for anti-HBc alone (OR, 2.6; P < 0.01). However, a significant OR was not obtained among the patients with a transient HBV infection implied by positivity for both antibody to hepatitis B surface antigen and anti-HBc (OR, 1.5; P = 0.48). These results indicate that past exposure to HBV is a risk factor for HCC in Japanese CLD patients, especially when they have no serological evidence of immunity to HBV.


*British Journal of Cancer* (1998) **77**(11), 2028–2031
© 1998 Cancer Research Campaign

### Past exposure to hepatitis B virus as a risk factor for hepatocellular carcinoma in patients with chronic liver disease

**S Okada[1], T Sato[2], T Okusaka[1], H Ishii[1], M Ikeda[1], H Nakasuka[1], H Kosakamoto[1], M Yoshimori[1] and K Wakabayashi[3]**

[1]Department of Internal Medicine, National Cancer Center Hospital, 5-1-1 Tsukiji, Chuo-ku, Tokyo 104, Japan; [2]Institute of Statistical Mathematics, 4-6-7 Minami-Azabu, Minato-ku, Tokyo 106, Japan; [3]Cancer Prevention Division, National Cancer Center Research Institute, 5-1-1 Tsukiji, Chuo-ku, Tokyo 104, Japan

**Summary** The aim of the study was to determine whether past exposure to hepatitis B virus (HBV) influences the risk of the development of hepatocellular carcinoma (HCC) in Japanese patients with chronic liver disease (CLD). We conducted a hospital-based case-control study of 141 HCC patients with CLD and 151 controls with CLD but without HCC. Past exposure to HBV was assessed by antibody to hepatitis B core antigen (anti-HBc) positivity. Ninety-two patients (65%) with HCC were anti-HBc positive compared with 65 patients (43%) with CLD alone ($P < 0.01$). A multivariate analysis using logistic regression modelling revealed that anti-HBc positivity significantly increased the risk of the development of HCC [odds ratio (OR) 2.0, $P = 0.01$]. In the anti-HBc-positive patients, a significantly increased risk of HCC was seen among the patients positive for anti-HBc alone (OR, 2.6; $P < 0.01$). However, a significant OR was not obtained among the patients with a transient HBV infection implied by positivity for both antibody to hepatitis B surface antigen and anti-HBc (OR, 1.5; $P = 0.48$). These results indicate that past exposure to HBV is a risk factor for HCC in Japanese CLD patients, especially when they have no serological evidence of immunity to HBV.

**Keywords:** hepatocellular carcinoma; chronic liver disease; hepatitis B virus; hepatitis C virus; case–control study

In Japan, the vast majority of patients with hepatocellular carcinoma (HCC) have chronic liver disease (CLD) such as chronic hepatitis and liver cirrhosis, and CLD patients frequently develop HCC during the follow-up period; patients with CLD are at increased risk of developing HCC (Ikeda et al, 1993; Tsukuma et al, 1993). Many CLD patients are, therefore, followed up periodically using ultrasonography (US) and serum α-fetoprotein (AFP) measurements to detect HCC at an early stage (Tanaka et al, 1990). Evaluation of the risk factors for HCC in CLD patients is imperative. Identification of the individuals who are at higher risk of HCC would contribute to the early diagnosis of this disease and to effective implementation of strategies for chemoprevention.

Numerous epidemiological and biological studies have indicated that a chronic infection with hepatitis B virus (HBV) and/or hepatitis C virus (HCV) plays an important role in hepatocarcinogenesis (Bruix et al, 1989; Saito et al, 1990; Kim et al, 1991). As for HBV, serum hepatitis B surface antigen (HBsAg) positivity, which indicates a chronic HBV carrier state, is a well-established risk factor for HCC (Beasley et al, 1981; Chen et al, 1991). However, only a few reports have addressed the relationship between past exposure to HBV and HCC and, as yet, no definite conclusion has been established (Chiba et al, 1996; Yu et al, 1997).

This hospital-based case–control study was conducted to evaluate whether past exposure to HBV, which was assessed by antibody to hepatitis B core antigen (anti-HBc) positivity, influenced the risk of developing HCC in Japanese CLD patients.

*Received 9 September 1997*
*Revised 8 December 1997*
*Accepted 18 December 1997*

*Correspondence to:* S Okada

### PATIENTS AND METHODS

### Study population

The HCC patient group comprised 141 consecutive patients with HCC and underlying CLD who were referred to the National Cancer Center Hospital, Tokyo, Japan, between January 1992 and December 1993. This group included 110 men and 31 women who had a mean age of 61.4 (range 25–81) years. The diagnosis of HCC was made by histological examination in 123 patients. In the remaining 18 patients, it was based on markedly elevated serum AFP levels (≥ 400 ng ml$^{-1}$) with space-occupying lesions demonstrable by various imaging studies, or on typical computerizd tomographic (CT) and/or angiographic findings.

The CLD control group comprised 151 patients with CLD but without evidence of HCC [96 men and 55 women, mean age 55.9 (range 25–79) years]. The CLD group consisted of consecutive referals during the same period as the HCC patients and the coexistence of HCC was ruled out by diagnostic methods such as US, CT and serum AFP measurements. In both groups, the diagnosis of CLD was based on biochemical evidence of liver parenchymal dysfunction and clinical features such as oesophageal varix and splenomegaly and, whenever possible, on histological examination (137 patients). Patients with specific types of CLD such as autoimmune hepatitis, primary and secondary biliary cirrhosis, and with CLD due to parasitosis, congestive heart failure or metabolic disorders were excluded because these CLDs appeared to differ substantially from the most common type of CLD observed in Japan.

### Laboratory studies

Blood specimens from all patients were processed shortly after collection and stored at – 70°C until analysis. All serum samples,

**Table 1** Distributions of age, gender, HBV markers, anti-HCV, history of blood transfusion and alcohol abuse in HCC patients and CLD patients

| Variable | No. of patients (%) | | Odds ratio (95% CI) | P-value |
|---|---|---|---|---|
| | HCC patients (n = 141) | CLD patients (n = 151) | | |
| Age | | | | |
| < 60 years | 54 (38) | 87 (58) | 1.0 | < 0.01 |
| ≥ 60 years | 87 (62) | 64 (42) | 2.2 (1.3–3.6) | |
| Gender | | | | |
| Female | 31 (22) | 55 (36) | 1.0 | 0.01 |
| Male | 110 (78) | 96 (64) | 2.0 (1.2–3.5) | |
| HBsAg | | | | |
| No | 118 (84) | 138 (91) | 1.0 | 0.07 |
| Yes | 23 (16) | 13 (9) | 2.1 (1.0–4.5) | |
| Anti-HBs | | | | |
| No | 125 (89) | 131 (87) | 1.0 | 0.75 |
| Yes | 16 (11) | 20 (13) | 0.8 (0.4–1.8) | |
| Anti-HBc | | | | |
| No | 49 (35) | 86 (57) | 1.0 | < 0.01 |
| Yes | 92 (65) | 65 (43) | 2.5 (1.5–4.1) | |
| Anti-HCV | | | | |
| No | 28 (20) | 44 (29) | 1.0 | 0.09 |
| Yes | 113 (80) | 107 (71) | 1.7 (0.9–3.0) | |
| History of blood transfusion | | | | |
| No | 86 (63) | 82 (56) | 1.0 | 0.25 |
| Yes | 50 (37) | 65 (44) | 0.7 (0.4–1.2) | |
| Alcohol abuse[a] | | | | |
| No | 93 (67) | 105 (69) | 1.0 | 0.72 |
| Yes | 46 (33) | 46 (31) | 1.1 (0.7–1.9) | |

[a]Ethanol intake ≥ 80 g day$^{-1}$ for ≥ 5 years

**Table 2** Distributions of age, gender, HBV markers, anti-HCV, history of blood transfusion and alcohol abuse in HBsAg-negative patients

| Variable | No. of patients (%) | | Odds ratio (95%) | P-value |
|---|---|---|---|---|
| | HCC patients (n = 118) | CLD patients (n = 138) | | |
| Age | | | | |
| < 60 years | 37 (31) | 75 (54) | 1.0 | < 0.01 |
| ≥ 60 years | 81 (69) | 63 (46) | 2.6 (1.5–4.5) | |
| Gender | | | | |
| Female | 25 (21) | 51 (37) | 1.0 | < 0.01 |
| Male | 93 (79) | 87 (63) | 2.8 (1.2–4.0) | |
| Anti-HBs | | | | |
| No | 103 (87) | 118 (85) | 1.0 | 0.82 |
| Yes | 15 (13) | 20 (15) | 0.9 (0.4–1.9) | |
| Anti-HBc | | | | |
| No | 49 (41) | 86 (62) | 1.0 | < 0.01 |
| Yes | 69 (59) | 52 (38) | 2.3 (1.4–4.0) | |
| Anti-HCV | | | | |
| No | 9 (8) | 34 (25) | 1.0 | < 0.01 |
| Yes | 109 (92) | 104 (75) | 4.0 (1.7–9.4) | |
| History of blood transfusion | | | | |
| No | 70 (62) | 73 (54) | 1.0 | 0.29 |
| Yes | 43 (38) | 61 (46) | 0.7 (0.4–1.3) | |
| Alcohol abuse[a] | | | | |
| No | 76 (65) | 96 (70) | 1.0 | 0.58 |
| Yes | 40 (35) | 42 (30) | 1.2 (0.7–2.1) | |

[a]Ethanol intake ≥ 80 g day$^{-1}$ for ≥ 5 years which were coded without regard to case or control status, were tested for HBsAg, antibody to HBsAg (anti-HBs), anti-HBc and antibody to HCV (anti-HCV). HBsAg was detected by reverse passive haemagglutination. Anti-HBs was measured by passive haemagglutination. Anti-HBc was evaluated by an enzyme immunoassay (EIA). Titres for anti-HBc showing more than 70% inhibition were assessed as positive, and these positive samples were further tested at 1:200 dilution. The determination of anti-HCV in serum samples was made using a second-generation EIA test.

### Statistical methods

The risk of developing HCC, related to each study variable, was evaluated by calculating the odds ratio (OR) with 95% confidence intervals (CI). A multivariate analysis was also performed by modelling the data through unconditional logistic regression for controlling possible confounding factors. Variables included in the model were age, gender, HBV markers (HBsAg, anti-HBs and anti-HBc), anti-HCV status, history of blood transfusion and alcohol abuse (ethanol intake ≥ 80 g day$^{-1}$ for ≥ 5 years). Adjusted ORs and 95% CI were derived from logistic regression coefficients.

Among the anti-HBc-positive patients, the ORs were calculated for subgroups of patients, defined by HBV markers, by using patients without any HBV markers as reference. In each subgroup, the proportion of patients showing a high inhibition rate (≥ 90%) at a 1 : 200 serum dilution in the anti-HBc assay was also examined.

Statistical analyses were performed using PC-SAS, version 6.09. Significance was defined as a *P*-value ≤ 0.05. All *P*-values quoted are two-sided.

### RESULTS

HBsAg was detected in 23 of the 141 HCC patients (16%) and 13 of the 151 CLD patients (9%) ($P = 0.07$); anti-HCV seropositivity was 80% and 71% in these two groups respectively ($P = 0.09$; Table 1). Concomitant chronic infection of HBV and HCV, indicated by the presence of both HBsAg and anti-HCV, was seen in four patients (3%) with HCC and three (2%) with CLD alone. Anti-HBc was positive in 92 HCC patients (65%) and 65 CLD patients (43%) ($P < 0.01$). Although the prevalence of anti-HBs was similar between the HCC and CLD groups ($P = 0.75$), most patients who were positive for anti-HBs were also positive for anti-HBc; only three HCC patients and six CLD patients were positive for anti-HBs alone. The univariate analysis revealed that the risk of HCC was strongly associated with age greater than 60 years (OR 2.2, $P < 0.01$), male gender (OR 2.0, $P = 0.01$) and anti-HBc positivity (OR 2.5, $P < 0.01$). The anti-HBs positivity, history of blood transfusion and alcohol abuse were not significantly different between the CLD patients with or without HCC.

Table 2 presents the distributions of the variables described above in the HBsAg-negative patients (118 HCC patients and 138 CLD patients). The prevalence of anti-HCV and anti-HBc was high in both the HCC patients and CLD patients; however, the prevalence of anti-HCV and anti-HBc was significantly higher in the HCC patients compared with patients who had CLD alone. Anti-HCV

© Cancer Research Campaign 1998

Table 3 Risk factors for HCC in CLD patients calculated by logistic regression analysis

| Variable | Odds ratio (95% CI) | P-value |
|---|---|---|
| Advancing age (by 10 years) | 1.9 (1.4–2.5) | < 0.01 |
| Male gender | 2.0 (1.1–3.5) | 0.02 |
| HBsAg positivity | 4.7 (1.7–12.7) | < 0.01 |
| Anti-HBc positivity | 2.0 (1.2–3.5) | 0.01 |
| Anti-HCV positivity | 2.3 (1.1–4.9) | 0.03 |

was detected in 109 HCC patients (92%) and in 104 CLD patients (75%) (OR 4.0, $P < 0.01$). Anti-HBc was detected in 69 HCC patients (59%) and in 52 CLD patients (38%) (OR 2.3, $P < 0.01$). In addition, significant ORs were obtained for age greater than 60 years (OR 2.6, $P < 0.01$) and male gender (OR 2.8, $P < 0.01$).

The results of the multivariate unconditional logistic regression analysis of risk factors are shown in Table 3. Advancing age by 10 years (OR 1.9, $P < 0.01$), male gender (OR 2.0, $P = 0.02$), HBsAg positivity (OR 4.7, $P < 0.01$), anti-HBc positivity (OR 2.0, $P = 0.01$) and anti-HCV positivity (OR 2.3, $P = 0.03$) were significantly related to the development of HCC. Anti-HBs positivity, history of blood transfusion and alcohol abuse were not found to be significantly associated with HCC and we did not, therefore, include them in the final model.

Table 4 presents the distribution of the patients defined by HBV markers in the anti-HBc-positive patients (92 HCC patients and 65 CLD patients). Anti-HBc positivity occurred in conjunction with three patterns of HBV markers: (1) with HBsAg indicating a persistent HBV infection (36 patients), (2) with anti-HBs indicating a resolved HBV infection (26 patients) and (3) without either of these two markers (95 patients). Anti-HBc was present in all 36 HBsAg positive patients, and among them, 30 (83%) demonstrated a high inhibition rate (≥ 90%) at a 1:200 serum dilution. However, all but two of the 121 HBsAg-negative patients who were positive for anti-HBc revealed a low inhibition rate (< 90%), indicating a previous transient HBV infection. The significantly increased risk of HCC was seen only among the patients positive for anti-HBc alone (OR 2.6, $P < 0.01$), and not among the patients positive for both anti-HBs and anti-HBc (OR 1.5, $P = 0.48$).

### DISCUSSION

Abundant evidence exists in support of the contribution of chronic HBV infection to HCC development. However, an aetiological role of transient HBV infection in hepatocarcinogenesis remains to be elucidated (Beasley et al, 1981; Chen et al, 1991; Ikeda et al, 1993; Tsukuma et al, 1993). In this study, we performed a case–control investigation of Japanese CLD patients to assess the influence of past exposure to HBV in the development of HCC. Past exposure to HBV was assessed by anti-HBc positivity, since anti-HBc is the most sensitive test available to detect a history of HBV infection.

We found that anti-HBc was more prevalent among the CLD patients with HCC compared with the CLD patients without HCC. Among only the HBsAg-negative patients, we also found a significantly higher prevalence of anti-HBc in the HCC patients. The multivariate analysis showed the same result regarding the role of anti-HBc as a risk factor for developing HCC. Moreover, in the anti-HBc-positive patients, the increased risk of HCC was seen among the patients positive for anti-HBc alone. All but two of the 95 patients positive for anti-HBc alone demonstrated a low inhibition rate at a 1:200 serum dilution, supporting the involvement of a previous transient HBV infection in the aetiology of HCC with CLD. However, the risk of HCC among the patients with a resolved HBV infection indicated by positivity for both anti-HBs and anti-HBc was not significantly enclosed. Anti-HBc positivity had never been shown to be an independent risk factor for HCC among CLD patients, although a significant relationship between HBV antibodies positivity and HCC has been reported both in the case–control study of HCC patients and control subjects without CLD (Yu et al, 1997) and in patients with HCV-related liver cirrhosis (Chiba et al, 1996).

The mechanism by which anti-HBc positivity is related to HCC remains to be elucidated, although a possible involvement of HBV in the carcinogenesis has been pointed out in HCC patients positive for anti-HBc (Maupas et al, 1975). There are at least two possible explanations for the close relationship between anti-HBc positivity and HCC:

(1) HBV can cause HCC by transient infection, even if the patients were not known to have chronic HBV infection. HBV-DNA may have been inserted into cellular DNA at an earlier stage, when the *X* gene or a truncated pre*S*/*S* gene may have been responsible for initiating tumorigenesis (Galloway and McDougall, 1983; Kim et al, 1991).
(2) Chronic HBV infection cannot be excluded in HBsAg-negative patients positive for anti-HBc, because it has been reported that HBV-DNA can be detected in their liver tissue and serum (Galloway and McDougall, 1983; Bréchot et al, 1985; Paterlini et al, 1990; Sheu et al, 1992). Further studies focusing on the analysis of HBV-DNA in the liver and serum are needed to clarify this point.

Table 4 Distribution of the patients defined by HBV markers in the anti-HBC-positive patients

| (HBsAg/anti-HBs/anti-HBc) | No. of patients | | | High inhibition rate[a] | Odds ratio (95% CI) | P-value |
|---|---|---|---|---|---|---|
| | HCC patients | CLD patients | Total | | | |
| (+/–/+) | 23[b] | 13 | 36 | 30 (83%) | 3.1 (1.3–7.2) | < 0.01 |
| (–/+/+) | 12 | 14 | 26 | 0 (0%) | 1.5 (0.6–3.8) | 0.48 |
| (–/–/+) | 57 | 38 | 95 | 2 (2%) | 2.6 (1.5–4.7) | < 0.01 |
| (–,–,–) | 46 | 80 | 126 | – | 1.0 – | – |

[a]More than or equal to 90% at a 1:200 serum dilution in the anti-HBc assay; [b]One patient was positive for both HBsAg and anti-HBs.

 © Cancer Research Campaign 1998

In conclusion, a significant relationship between anti-HBc status and HCC was revealed in the Japanese CLD patients. Therefore, the determination of anti-HBc status might contribute to the identification of patients at higher risk for HCC, and to the optimization of the timing and frequency of follow-up programmes. CLD patients positive for anti-HBc (irrespective of HBsAg status) should be followed more closely for the early detection of HCC, especially when they have no serological evidence of immunity to HBV. However, long-term follow-up studies of CLD patients should provide additional evidence helpful in defining more precisely and directly the magnitude of HCC risk associated with anti-HBc. Further studies are also mandatory to establish whether our findings are consistent with other geographical areas where the distribution of HBV and HCV is substantially different.

### ACKNOWLEDGEMENTS

This study was supported in part by a Grant-in-Aid for Cancer Research from the Ministry of Health and Welfare of Japan. We wish to thank Ms Keiko Kondo for help with manuscript preparation.

### REFERENCES

Beasley RP, Hwang LY, Lin CC and Chien CS (1981) Hepatocellular carcinoma and hepatitis B virus. A prospective study of 22 707 men in Taiwan. *Lancet* **ii**: 1129–1133

Bréchot C, Degos F, Lugassy C, Thiers V, Zafrani S, Franco D, Bismuth H, Trépo C, Benhamou JP, Wands J, Isselbacher K, Tiollais P and Berthelot P (1985) Hepatitis B virus DNA in patients with chronic liver disease and negative tests for hepatitis B surface antigen. *N Engl J Med* **312**: 270–276

Bruix J, Barrera JM, Calvet X, Ercilla G, Costa J, Sanchez-Tapias JM, Ventura M, Vall M, Bruguera M, Bru C, Castillo R and Rodes J (1989) Prevalence of antibodies to hepatitis C virus in Spanish patients with hepatocellular carcinoma and hepatic cirrhosis. *Lancet* **ii**: 1004–1006

Chen CJ, Liang KY, Chang AS, Chang YC, Lu SN, Liaw YF, Chang WY, Sheen MC and Lin TM (1991) Effects of hepatitis B virus, alcohol drinking, cigarette smoking and familial tendency on hepatocellular carcinoma. *Hepatology* **13**: 398–406

Chiba T, Matsuzaki Y, Abei M, Shoda J, Aikawa T, Tanaka N and Osuga T (1996) Multivariate analysis of risk factors for hepatocellular carcinoma in patients with hepatitis C virus-related liver cirrhosis. *J Gastroenterol* **31**: 552–558

Galloway DA and McDougall JK (1983) The oncogenic potential of herpes simplex viruses: evidence for a 'hit-and-run' mechanism. *Nature* **302**: 21–24

Ikeda K, Saitoh S, Koida I, Arase Y, Tsubota A, Chayama K, Kumada H and Kawanishi M (1993) A multivariate analysis of risk factors for hepatocellular carcinogenesis: a prospective observation of 795 patients with viral and alcoholic cirrhosis. *Hepatology* **18**: 47–53

Kim CM, Koike K, Saito I, Miyamura T and Jay G (1991) HBX gene of hepatitis B virus induces liver cancer in transgenic mice. *Nature* **351**: 317–320

Maupas P, Werner B, Larouzé B, Millman I, London WT, O'Connell A and Blumberg BS (1975) Antibody to hepatitis-B core antigen in patients with primary hepatic carcinoma. *Lancet* **ii**: 9–11

Paterlini P, Gerken G, Nakajima E, Terre S, D'Errico A, Grigioni W, Nalpas B, Franco D, Wands J, Kew M, Pisi E, Tiollais P and Bréchot C (1990) Polymerase chain reaction to detect hepatitis B virus DNA and RNA sequences in primary liver cancers from patients negative for hepatitis B surface antigen. *N Engl J Med* **323**: 80–85

Saito I, Miyamura T, Ohbayashi A, Harada H, Katayama T, Kikuchi S, Watanabe Y, Koi S, Onji M, Ohta Y, Choo QL, Houghton M and Kuo G (1990) Hepatitis C virus infection is associated with the development of hepatocellular carcinoma. *Proc Natl Acad Sci USA* **87**: 6547–6549

Sheu JC, Huang GT, Shih LN, Lee WC, Chou HC, Wang JT, Lee PH, Lai MY, Wang CY, Yang PM, Lee HS and Chen DS (1992) Hepatitis C and B viruses in hepatitis B surface antigen-negative hepatocellular carcinoma. *Gastroenterology* **103**: 1322–1327

Tanaka S, Kitamura T, Nakanishi K, Okuda S, Yamazaki H, Hiyama T and Fujimoto I (1990) Effectiveness of periodic checkup by ultrasonography for the early diagnosis of hepatocellular carcinoma. *Cancer* **66**: 2210–2214

Tsukuma H, Hiyama T, Tanaka S, Nakao M, Yabuuchi T, Kitamura T, Nakanishi K, Fujimoto I, Inoue A, Yamazaki H and Kawashima T (1993) Risk factors for hepatocellular carcinoma among patients with chronic liver disease. *N Engl J Med* **328**: 1797–1801

Yu MC, Yuan JM, Ross RK and Govindarajan S (1997) Presence of antibodies to the hepatitis B surface antigen is associated with an excess risk for hepatocellular carcinoma among non-Asians in Los Angeles County, California. *Hepatology* **25**: 226–228

© Cancer Research Campaign 1998